# Estimating the number of secondary Ebola cases resulting from an unsafe burial and risk factors for transmission during the West Africa Ebola epidemic

**DOI:** 10.1371/journal.pntd.0005491

**Published:** 2017-06-22

**Authors:** Amanda Tiffany, Benjamin D. Dalziel, Hilary Kagume Njenge, Ginger Johnson, Roselyn Nugba Ballah, Daniel James, Abdoulaye Wone, Juliet Bedford, Amanda McClelland

**Affiliations:** 1 Epicentre, Geneva, Switzerland; 2 Department of Integrative Biology, Oregon State University, Corvallis, Oregon, United States of America; 3 Department of Mathematics, Oregon State University, Corvallis, Oregon, United States of America; 4 International Federation of the Red Cross and Red Crescent Societies, Monrovia, Liberia; 5 Anthrologica, Dallas, Texas, United States of America; 6 Liberian Red Cross, Monrovia, Liberia; 7 Sierra Leone Red Cross Society, Freetown, Sierra Leone; 8 International Federation of the Red Cross and Red Crescent Societies, Conakry, Guinea; 9 Anthrologica, Oxford, United Kingdom; 10 International Federation of the Red Cross and Red Crescent Societies, Geneva, Switzerland; Common Heritage Foundation, NIGERIA

## Abstract

**Background:**

Safely burying Ebola infected individuals is acknowledged to be important for controlling Ebola epidemics and was a major component of the 2013–2016 West Africa Ebola response. Yet, in order to understand the impact of safe burial programs it is necessary to elucidate the role of unsafe burials in sustaining chains of Ebola transmission and how the risk posed by activities surrounding unsafe burials, including care provided at home prior to death, vary with human behavior and geography.

**Methodology/Principal findings:**

Interviews with next of kin and community members were carried out for unsafe burials in Sierra Leone, Liberia and Guinea, in six districts where the Red Cross was responsible for safe and dignified burials (SDB). Districts were randomly selected from a district-specific sampling frame comprised of villages and neighborhoods that had experienced cases of Ebola. An average of 2.58 secondary cases were potentially generated per unsafe burial and varied by district (range: 0–20). Contact before and after death was reported for 142 (46%) contacts. Caregivers of a primary case were 2.63 to 5.92 times more likely to become EVD infected compared to those with post-mortem contact only. Using these estimates, the Red Cross SDB program potentially averted between 1,411 and 10,452 secondary EVD cases, reducing the epidemic by 4.9% to 36.5%.

**Conclusions/Significance:**

SDB is a fundamental control measure that limits community transmission of Ebola; however, for those individuals having contact before and after death, it was impossible to ascertain the exposure that caused their infection. The number of infections prevented through SDB is significant, yet greater impact would be achieved by early hospitalization of the primary case during acute illness.

## Author summary

The care of an individual infected with Ebola virus disease (EVD), their death, funeral, and burial in the community rather than in an Ebola Treatment Center (ETC) poses a serious risk for continued disease transmission. Consequently, SDB is an essential component of EVD outbreak response; however, its impact on transmission is not well understood. During the 2013–2016 EVD epidemic the Red Cross carried out over 50% of the official burials in Guinea, Liberia and Sierra Leone. We performed epidemiological investigations in EVD affected communities to better understand disease transmission linked to unsafe burials of (suspect) EVD infected individuals, and risk factors for transmission linked to caring and burial practices. An average of 2.58 secondary cases were potentially generated per unsafe burial investigated and varied by district (range: 0–20). Additionally, the Red Cross SDB program potentially averted between 1,411 and 10,452 secondary EVD cases, reducing the epidemic by 4.9% to 36.5%. Our results quantify for the first time the potential impact this essential EVD response component had on the 2013–2016 epidemic and highlight the importance of SDB as a fundamental control measure, while also underlining the well-known importance of isolating EVD infected individuals as soon as they show symptoms in order to limit transmission.

## Introduction

The 2013–2016 West Africa Ebola virus disease epidemic reached a scale never seen before. Over 28,600 people were infected with Ebola virus disease (EVD) and over 11,000 died [1,2]. Any activity involving direct, unprotected contact between living individuals and an EVD infected individual, living or deceased, are potential mechanisms for onward transmission of the virus. Transmission of EVD occurs when an uninfected individual has direct contact with the blood or body fluids of an EVD infected, symptomatic individual or objects contaminated with their blood or body fluids. Risk of EVD transmission is particularly high during caregiving practices and when bodies are being prepared for funeral rites [3,4]. Consequently, relatives, community members and healthcare workers without appropriate personal protective equipment have an increased risk of infection.

Despite common modes of transmission, the dynamics of the 2013–2016 EVD epidemic varied widely between and within the most affected countries of Guinea, Liberia and Sierra Leone [5,6]. Nevertheless key pillars of the response were systematically implemented across all affected countries. These pillars, common to EVD outbreaks, focused on preventing disease transmission through case management in an Ebola Treatment Center (ETC), contact tracing, social mobilization and safe burials of EVD infected bodies [7,8].

Deaths of EVD positive individuals that occur in the community, outside an ETC, pose a serious risk for continued EVD transmission. The infection status of the deceased is frequently unknown at the time of death and there are many opportunities for contact with the deceased. These can result, for example, from local customs including washing, redressing, and burial of the body [9,10]. When an EVD positive individual dies in the community, both familial abstention from contact with the deceased, and a safe and dignified burial (SDB) carried out by a trained team, are necessary to prevent burial- and funeral-related transmission. When an EVD infected body is buried safely, onward transmission of the virus as a consequence of burial or funeral rituals should cease. A SDB, in the strictest terms, should result in no direct contact with the infected body after the time of death. Consequently, no secondary cases should result from a safe burial, and onward transmission should be limited.

The importance of SDB as an integral part of reducing EVD transmission and stopping an outbreak is accepted, but not well understood. While work has been conducted to ascertain the impact of individual EVD interventions on disease transmission, and the importance of community deaths in secondary case generation, most of the data come from limited, focal case time series or relies on data on cases and deaths as published by the World Health Organization (WHO) [11–14]. Until this study, neither research focusing specifically on safe burials nor quantification of the direct impact of such activities had been conducted. Using data collected during epidemiological investigations, we estimate the number of secondary cases that were potentially averted by safe burials, and describe risk factors for EVD transmission during funerals and burial rituals (unsafe burials). The potential impact of the SDB program on the 2013–2016 EVD epidemic as a result of activities carried out by the National Societies of the Red Cross in Sierra Leone, Guinea and Liberia, supported by the International Federation of the Red Cross and Red Crescent Societies (IFRC), is also estimated.

## Methods

### Ethics statement

Authorization to conduct this research was obtained from the Sierra Leone Ministry of Health and Sanitation, the Guinea Ministry of Health and Public Hygiene and the Liberia Ministry of Health and Social Welfare. Informed consent forms were read and explained to each research participant. Due to restrictive infection prevention procedures, consent to participate was given orally in order to avoid contact between the research team and the key informants. Consent to participate was noted by the research team on the consent form. Participation was voluntary and it was made clear that consent could be withdrawn by the participant at any time and for any reason without repercussion.

All epidemiological data were anonymized before being entered into data spreadsheets. All research documents were stored in a locked cabinet or electronically on a password-protected computer with access available only to the research team. Data back-ups were made on external support.

### Study setting and population

The Red Cross conducted approximately 50% of all official SDBs in Sierra Leone, 100% in Guinea and 100% in Montserrado County, Liberia during the 2013–2016 epidemic. Data collection was carried out in rural and urban districts in Sierra Leone, Guinea and Liberia where the National Society in each country, supported by the IFRC (hereafter referred to as the Red Cross) were responsible for SDB, as depicted in Table 1 [15–17].

**Table 1:** Population and area estimates for the districts where unsafe burials were investigated.

Country	District*	Population^+^	Area	First EVD Case
Sierra Leone	Western Area Rural	197,098	544 km^2^	August 2014
Sierra Leone	Kambia	313,765	3,108 km^2^	September 2014
Sierra Leone	Kailahun	409,520	3,859 km^2^	April 2014
Guinea	Guéckédou	405,000	4,400 km^2^	December 2013
Guinea	Forécariah	136,000	4,200 km^2^	September 2014
Liberia	Montserrado	1,200,000	1,909 km^2^	June 2014

*The generic term “district” is used to refer to the different administrative divisions in the three countries. The appropriate nomenclature for each country is: Guinea (prefecture), Liberia (county), Sierra Leone (district).

^+^Population calculations are based on 2004 data with an annual 6% growth rate.

### Implementation

Data collection was carried out from June 18, 2015 to August 4, 2015. Interviews were conducted for four days in each district, except for Montserrado County where they were conducted for eight days. To the furthest extent possible, attempts were made to inform the leaders of the selected villages or urban neighborhoods (hereafter referred to as community)of the arrival of the research team at least one day prior to their visit in order to ensure the availability of key informants and receive the necessary approvals from the village chief and community leaders.

Prior to data collection in each district, a member of the local Red Cross who was familiar with the area and able to translate between English and the local language(s) was trained on the research methodology and data collection tool. Together with the study’s epidemiologist, they visited each community selected and met with community leaders and key informants. Key informants were identified by the Red Cross volunteer in collaboration with community members. One primary key informant was purposively selected in each community to provide information on the burial of interest. This person was generally a family member of the deceased or if a relative was no longer present, another individual identified by the community.

### Safety

Data collection occurred during periods of active EVD transmission in some districts. All activities were therefore conducted in accordance with strict infection prevention procedures that focused on avoiding contact between the data collection team, key informants and other community members. All members of the data collection team were provided with personal protection equipment while in the field including gum (rubber) boots, chlorine spray bottles and hand sanitizer containing at least 70% alcohol. In addition, team members were given daily reminders on safety procedures to adopt while in the field to ensure their safety.

### Sampling frame

The sampling frame was comprised of communities (rural villages and urban neighborhoods) in priority districts identified by the Red Cross that had experienced at least one of the following events: a) cases of EVD; b) community deaths during the period of the epidemic; or c) having a community member hospitalized in an ETC. If this information was unavailable, a list of all communities in the selected district was used in its place.

During selection, one group of 10 communities (G1) was randomly selected from the sampling frame. After communities were selected, key informants were identified in each community and used to ascertain: 1) if any cases of EVD had occurred in the community, and 2) if an unsafe burial of an EVD case had taken place. If the answer to both questions was yes, the community was included in the final list of communities to be visited by the study team. If at least five communities with unsafe burials were not found in G1, another group of 10 communities (G2) was selected from the sampling frame until at least five communities that reported an unsafe burial of an EVD infected individual were identified.

If the history of an unsafe burial was unable to be reconstructed due to loss of key informants in the community or another cause, another unsafe burial was chosen from the list. If more than one unsafe burial related to the same source case took place in the same community, data were collected from one of the burials, generally the first one that took place.

### Definitions

An unsafe burial was considered to be a burial of an individual with suspect EVD infection, buried by their community, family, or manipulated after death but prior to the arrival of the SDB team. The first identified suspect EVD case that was buried unsafely was considered to be the primary case for the purposes of further investigation. A contact was defined as any individual who had physical contact with the body of the deceased, their body fluids or their (potentially infected) belongings after death as reported by the key informant. A potential secondary case was defined as a contact that met either the baseline or ceiling definition (see below). These are called “potential” secondary cases because transmission was not directly observed, and it is therefore possible that some secondary cases acquired the infection from a source other than the primary case.

### Data collection

Data were collected on a standardized questionnaire, from key informants, regarding the deceased (primary case) and their contacts, by the epidemiologist in collaboration with the local Red Cross member(s). For each primary case, details of age, sex, source of infection, EVD swab confirmation and secondary cases in individuals listed as having had contact with the deceased (contacts) were collected. For each individual reported to have had contact with the deceased, age, sex, type of contact(s) with the body of the deceased, current status (alive/dead) and EVD swab confirmation was collected. In addition, details about family ties and relationship to the deceased were collected in Liberia and Guinea.

### Data management

Completed questionnaires were reviewed daily for completeness and data entered into a Microsoft Excel spreadsheet. Data analysis was conducted in Stata 12 (Stata Corp, College Station, TX) and R (R Foundation for Statistical Computing, Vienna, Austria).

### Data analysis

The average number of secondary infections potentially caused by an unsafe burial, and risks of secondary infection associated with different types of contact were estimated using two datasets, both extracted from the epidemiological data collected in the field. The first dataset from which results for the baseline estimate were produced consisted of only EVD positive cases (as reported by the key informant), and contacts were limited to those that only had contact with the primary case after death (i.e. caregivers were excluded). The second dataset from which results for the ceiling estimate were produced consisted of all data collected and included anyone who became sick or died following contact with the primary case (before or after death) as reported by the key informant. The number of secondary cases potentially averted was calculated as the average number of potential secondary cases per unsafe burial multiplied by the number of EVD positive burials carried out by the Red Cross. The number of SDB carried out by Red Cross SDB teams, by country, is presented as reported by the Red Cross.

The number of SDB that were laboratory positive corresponds to the number of deaths reported from October 2014 to April 2015 in Sierra Leone for which laboratory results were available and matched based on name, age and sex using a computer algorithm (~75% success in matching) in addition to all positive community deaths in Guinea and Montserrado County, Liberia.

Pooled and district specific estimates are presented with medians and interquartile ranges. Estimates of risk factors among contacts of a primary case are presented as odds ratios based on two-sided Fisher’s exact test and presented with 95% confidence intervals. In order to construct the “cared for” odds ratio, the baseline case definition was altered to include caregivers otherwise the case definitions for the baseline and ceiling estimates remained the same for the risk analysis, with the baseline estimate excluding caregivers.

## Results

Forty-five unsafe community burials were found and investigated (Table 2). In three of the 42 communities (one in Forécariah, Guinea, and two in Montserrado, Liberia), two unsafe burials were investigated. Two unsafe burials were reported to have not resulted in secondary cases (both in Sierra Leone, one in Kambia and Kailahun). At least five unsafe burials were documented in each of the six districts except for Forécariah (Guinea) where only three unsafe burials were investigated.

**Table 2:** Description of reported primary case contacts as described by key informants by country and district.

	Sierra Leone			Guinea		Liberia	Overall
	Western Area Rural	Kambia	Kailahun	Guéckédou	Forécariah	Montserrado	Total
**Communities included, N**	6	5	9	8	2	12	42
**Burials investigated, N**	6	5	9	8	3	14	45
**Contacts identified, N**	46	18	77	69	24	76	310
**Contacts per burial, median [range]**	8 [2–13]	2 [2–8]	8 [4–19]	11 [3–20]	5 [4–15]	5 [1–12]	7 [1–20]
**Age**^+^**, median [IQR]**	54 [20–48]	35 [30–50]	45 [35–55]	40 [33–55]	30 [23–46]	39 [29–55]	40 [30–40]
**Sex, % male**	50	72	56	75	58	54	60
**Health status known, n (% of N contacts identified)**	42 (91.3)	15 (83.4)	77 (100)	69 (100)	75 (98.7)	23 (95.9)	301 (97.1)
**Sick, n (% of N contacts identified)**	41 (89)	6 (33)	29 (38)	50 (72)	14 (58)	63 (83)	203 (65)
**Confirmed EVD infection, n (% of N contacts identified)**	33 (71)	5 (28)	7 (9)	4 (6)	5 (21)	24 (32)	78 (25)

*All percentages rounded to the nearest whole number; categorical variables will not always sum to 100%

^+^ Age was not reported for 57 individuals in Sierra Leone (Western Area Rural = 22, Kambia = 5 and Kailahun = 30)

Across the study, 310 individuals were identified and reported to have had contact with the body of the primary case after death, with an average of seven contacts per unsafe burial. The number of contacts reported from each unsafe burial ranged widely in and across the three countries: 2–19 in Sierra Leone 3–20 in Guinea and 1–12 in Liberia. Contacts were predominately male (60%) with a median age of 40 years (IQR: 30–40).

### Secondary cases associated with unsafe burials

Across the study, 203 contacts, 65% of those recorded, were reported to have become sick after having contact with the body of the primary case during an unsafe burial (Table 3). This ranged from 89% in Western Area Rural (Sierra Leone) to 33% in Kambia (Sierra Leone).

**Table 3:** Reported illness after contact with the primary case and timing of contact as reported by key informants.

Contact	Reported to have fallen sick after contact, (%)	
	No	Yes
**Before & after death**	22 (22)	120 (59)
**After death only**	76 (88)	83 (41)
**Total:**	98	203

Forty-one percent (83/203) of individuals that were reported to have fallen sick had contact with the primary case only after death; however, the key informant was not always aware if EVD infection had been confirmed by laboratory testing. The post-contact health status (sick or EVD confirmed) was unknown by key informants for nine contacts. For those whose health status was known, 25% (78/301) were reported to have fallen ill and have had a laboratory confirmed EVD infection (Table 2).

### Contact during unsafe burials

Investigations at the household level revealed that contact was not only limited to contact with the primary case after death. Contacts that occurred after death were classified into seven categories (Table 4). Key informants frequently reported that some individuals had more than one type of contact with the primary case.

**Table 4:** Types of contact with primary cases as reported by key informants by country and district.

	Sierra Leone			Guinea		Liberia	Overall
	Western Area Rural	Kambia	Kailahun	Guéckédou	Forécariah	Montserrado	Total
**Contacts identified, N**	46	18	77	69	24	76	310
**Care during illness, n (%)**	17 (37)	5 (28)	22 (29)	39 (57)	12 (50)	47 (62)	142 (46)
**Contact after death: n, (% N)**							
**Blood/body fluids**	0	0	4 (5)	1 (5)	6 (25)	10 (13)	21 (7)
**Washed clothes/bedding**	6 (13)	2 (11)	5 (6)	12 (17)	2 (8)	13 (17)	40 (13)
**Washed body**	18 (39)	5 (28)	40 (52)	21 (81)	7 (29)	21 (28)	112 (36)
**Transported body**	9 (20)	0	16 (21)	0	14 (58)	36 (47)	75 (24)
**Burial/funeral rituals**	2 (4)	0	14 (18)	18 (26)	11 (46)	41 (54)	86 (28)
**Burial of body**	19 (41)	12 (67)	25 (32)	11 (14)	11 (46)	32 (42)	110 (35)
**Other**	6 (13)	0	1 (1)	14 (54)	0	1 (1)	22 (7)
**Used protection, n (%)**	0	1 (6)	12 (16)	0	2 (8)	8 (11)	23 (7)

Individuals who had contact with the primary case after death were frequently reported (46%, 142/310) to have had contact with the same individual during the acute phase of their illness. Twenty-three contacts (7%, 23/310) were reported to have protected themselves when having contact with the primary case during acute illness or after death. Use of protection was most frequently reported in Kailahun (Sierra Leone) (16% of contacts, 12/77) and Montserrado County (Liberia) (11% of contacts, 8/76).

### Risk related to contact

**Fig 1:**
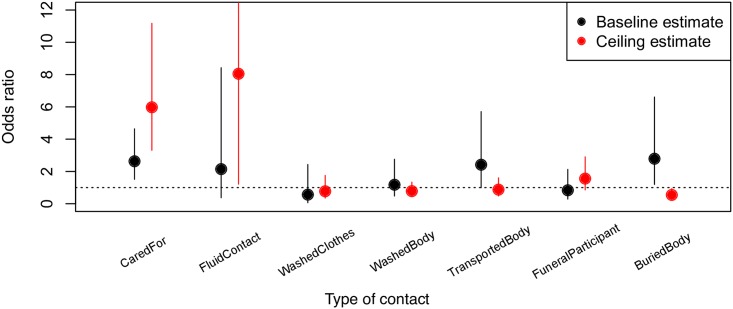
Estimates of risk factors among contacts of a primary case, in contributing to the chance of becoming a secondary case. Circles show the estimate for the odds ratio based on Fisher’s exact test. Vertical lines enclose the 95% confidence interval surrounding the estimate. In calculating the odds ratio for caregivers in the baseline estimate (where caregivers are normally excluded), the inclusion criteria for the baseline dataset were amended to include anyone who was EVD positive, instead of the standard baseline criteria of EVD positives who were not caregivers, since the standard baseline criteria would lead to no cases who were caregivers, by definition.

The exposure that was most strongly predictive of secondary transmission was having contact with fluids (e.g. blood, vomit) of a primary case, followed by direct physical contact with a primary case during their acute illness (Fig 1).

Overall, in the baseline estimate, 9.5% (+/- 1.6% s.e.; n = 45) of contacts became secondary cases, meaning that these contacts had exposures and outcomes that met the baseline case definition (not caregivers, and were reported to have become infected with EVD). In the ceiling estimate, 68% (+/- 2.6% s.e.; n = 45) of contacts became secondary cases, meaning that these contacts either became sick or died after their exposure.

In the baseline estimate, all laboratory confirmed EVD cases reported some form of contact after death. This precludes a finite estimate of the odds ratio associated with contact after death in the baseline estimate (the lower bound of the 95% confidence interval on the estimate was 8.0). In the ceiling estimate, the odds ratio was 0.16 (95% CI: 0.089–0.30). This reflects that many contacts in this estimate were considered EVD positive, but had contact both before and after death.

People who had contact with the primary case before death (caregivers) were, on average, between 2.63 (95% CI: 1.15–4.63, baseline estimate) and 5.97 (95% CI: 3.31–11.17, ceiling estimate) times more likely to become EVD infected, relative to people who only had contact with the primary case after death. For data on fluid contact, a small sample size caused the upper confidence bound to exceed 300, but this is a sampling artifact and should not be interpreted as containing information on risk patterns.

### Variation in estimates by place and time

**Fig 2:**
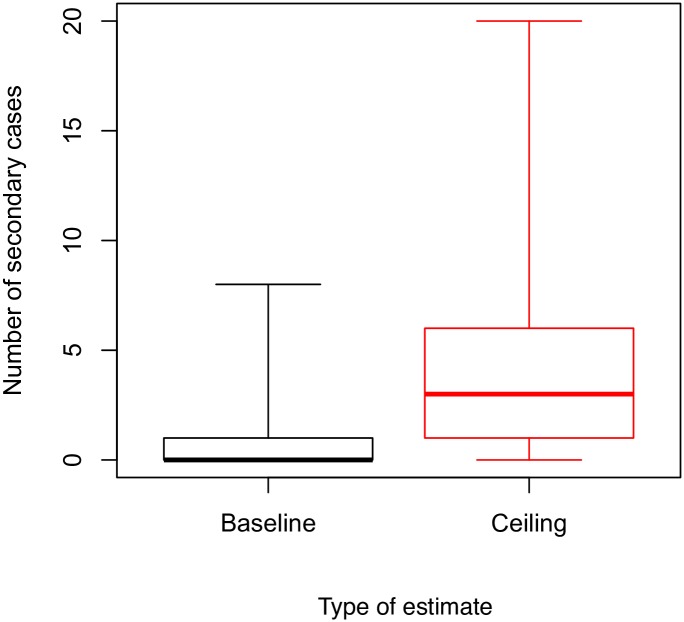
Estimates and uncertainty in the number of secondary cases caused by an unsafe burial, pooled across districts, for baseline and ceiling estimates. Thick horizontal lines show median estimate, boxes enclose the interquartile range in the estimate (between the 25^th^ and 75^th^ percentile) across primary cases and districts. Capped vertical lines encompass the total range of variability in the estimate.

**Fig 3:**
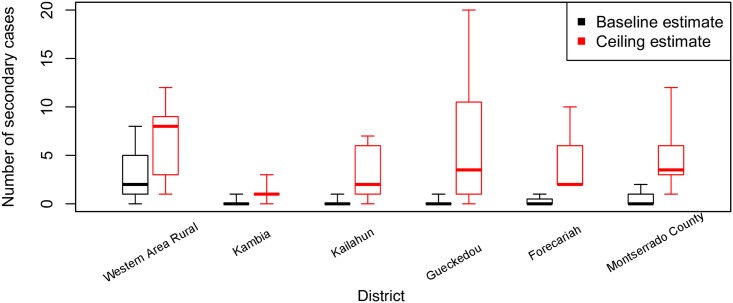
Estimates and uncertainty in the number of secondary cases caused by an unsafe burial, by district. Boxes enclose the interquartile range of each estimate (extending from the 25th percentile to the 75th percentile). The thick horizontal lines within each box correspond to the median. Capped vertical lines encompass the total range of variability in the estimate.

In the baseline estimate, a single unsafe burial was associated with an average of 0.64 (1.46 s.d., n = 45) secondary cases (Fig 2). This varied among districts from 0.125 in Guéckédou (Guinea) to 3.00 in Western Area Rural (Sierra Leone) (Fig 3). In the ceiling estimate, a single unsafe burial was associated with an average of 4.74 (4.27 s.d., n = 45) secondary cases with variation among districts ranging from 1.2 (1.09 s.d., n = 6) in Kambia (Sierra Leone), and 6.25 (7.39 s.d., n = 6) in Guéckédou (Guinea) (Fig 3). In both the baseline and ceiling estimates there was no statistically significant evidence of systematic variation among districts; just evidence of an abundance of variability, likely reflecting latent heterogeneities in transmission, and the stochastic nature of the underlying disease transmission process.

### Red Cross safe and dignified burial program impact

A total of 47,505 SDB were reported to have been carried out by the National Societies in Sierra Leone, Guinea and Liberia from 2013–2016. The number of burials completed varied greatly by country (Table 5). When laboratory results were received and matched with the individuals buried, 2,205 (4.6%) of the bodies buried tested positive for EVD in the laboratory.

**Table 5:** Number of safe and dignified burials carried out by the Red Cross by country and the number of those deaths that were EVD positive.

Country	Reported SDB completed	EVD positive
**Sierra Leone**	26,308	1,413
**Guinea**	17,513	254
**Liberia**	3,684	538
**Total:**	47,505	2,205

The relatively small number of bodies buried in Liberia, compared to Sierra Leone and Guinea, is related to the areas the Red Cross was responsible for SDB. In Sierra Leone and Guinea, the National Societies were responsible for burials in the entire country, while in Liberia the area of intervention was limited to Montserrado County only.

### EVD cases averted

**Fig 4:**
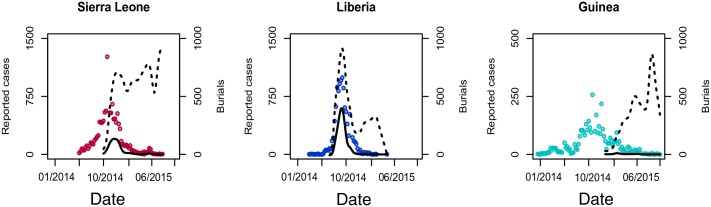
Reported cases in the WHO patient database and burials over time in Sierra Leone (left), Liberia (center) and Guinea (right). Colored dots show reported cases from the WHO patient database. Dotted lines show the total number of recorded burials carried out. Solid lines show estimates of the number of burials of EVD casualties, reconstructed from laboratory testing of swab samples collected from community deaths.

Fig 4 presents estimates of the total number of reported burials carried out by the Red Cross in addition to estimates of the number of these burials that were EVD positive, contrasted with the reported cases from the WHO patient database for Sierra Leone, Liberia and Guinea.

Applying the analysis presented above to the number of positive EVD cases buried, the number of secondary cases potentially caused by a typical unsafe burial leads to estimates for the number of secondary cases potentially averted by the SDB program. Using this method we estimate that between 1,411 (lower estimate) and 10,452 (upper estimate) secondary EVD cases may have been prevented by the SDB program. This represents a reduction in the total size of the 2013–2016 EVD epidemic of between 4.9% (lower estimate) and 36.5% (upper estimate). Thus, the total number of EVD cases in the 2013–2016 epidemic could have been 30,012 to 39,053 if the SDB program had not been implemented by the Red Cross in Sierra Leone, Guinea and Liberia.

## Discussion

The majority of the unsafe burials investigated seemed to be relatively representative of classic EVD transmission with between 0.64 to 4.74 secondary EVD cases associated with unsafe burials, an overall average of 2.58. Estimates of the average number of secondary cases potentially resulting from an unsafe burial are significant although there is variation depending on which estimate, baseline or ceiling, was used. This variation and the use of two estimates highlight the uncertainty surrounding the dynamics of EVD transmission. Additionally, both the number of individuals reported to have had contact with the primary case, and the type of contact they had during an unsafe burial, ranged widely by district. This variation may be due to many factors including the incidence of EVD in the district and the point in the epidemic during which the unsafe burial occurred. Accordingly, the highest median number of contacts per burial in this research was reported in Guéckédou (Guinea), the first district affected by the EVD epidemic, and the lowest in Kambia (Sierra Leone), the last of the sampled districts to be affected. This variation may reflect changes in behavior after exposure to EVD related messaging by Red Cross and other partners, and familiarity of individuals and communities with the disease.

The type of contact that was had with a primary case and the timing of that contact, before or after death, are important factors in EVD transmission. Such information is normally collected during field outbreak investigations for the purposes of contact tracing and to ascertain epidemiological links to known cases. In the early days of the EVD epidemic, analysis of contact tracing data showed that of 701 confirmed EVD cases 67 (10%) reported contact with EVD infected individuals after death (only), while 148 (21%) reported contact both before and after death [18]. Furthermore, some of the potential secondary cases identified in our study may have been infected by a source other than the primary case. In these cases, preventing exposure to the primary case would still reduce the risk of onward transmission, but would not be guaranteed to avert the secondary case altogether. Risk estimates from data presented here show, similar to previous studies, that having contact with the primary case both during illness and after death significantly increased the risk of EVD transmission compared to individuals who had contact with the primary case only after death [3,14,19]. Further analyses of risk factors for EVD transmission highlight the importance of having direct contact with the blood or body fluids of the deceased. Although our sample size was too small to provide a reliable estimate of risk due to such contact, exposure to blood and body fluids of the deceased was frequently cited by key informants and cannot be discounted as an important factor in transmission, as documented in previous risk factor analyses [3,19].

Reports of protective measures taken while having contact with a primary case varied in frequency and method, reflecting distinct differences in EVD awareness and behavior. Knowledge, attitudes and practices (KAP) surveys carried out at different points in the epidemic also highlighted geographic differences regarding EVD and its transmission [20,21]. Significant differences were noted in areas (defined differently between surveys) that had a higher incidence of EVD cases when compared to areas with lower EVD case incidence. While data collected through KAP surveys are subject to limitations, the differences between areas remains illustrative.

Not only are the processes that control changes in the number of individuals infected with EVD by place or over time not well understood, the number of individuals infected during the West African epidemic is also not certain due to significant underreporting [2,22]. Furthermore, neither burials carried out by other organizations during the epidemic, nor burials conducted early in the epidemic by the Red Cross are taken into consideration in this estimation. Systematic data collection by Red Cross burial teams only began later in the response (July 2014 in Liberia, October 2014 in Sierra Leone, and January 2015 in Guinea), laboratory data were difficult to obtain in some areas and data from other organizations involved in SDB were unavailable resulting in an underestimation of both overall SDB program impact and the impact of the Red Cross SDB program in particular.

The impact of an SDB program is highly dependent on disease prevalence at the time of program initiation. As seen in the data from Guinea, it appears that initiating SDB later in the epidemic when there are fewer EVD cases results in a dramatic reduction in program impact because the bodies buried will primarily be EVD negative. The Red Cross burial program began early in April 2014 in Guinea; yet, systematic collection of samples from the deceased only began in January 2015 when the prevalence of EVD was lower. As a result, the Red Cross program in Guinea is estimated to have buried mostly EVD negative bodies as reflected in the low numbers of confirmed laboratory positive bodies. This contrasts with Liberia where less than 20% of t burials were carried out by the Red Cross; however, twice the number of positive bodies were buried. This is likely a consequence of the high prevalence of EVD when SDB was initiated in Liberia, and an EVD positivity that was higher overall.

The impact of SDB on preventing chains of transmission was not investigated and may have been particularly relevant in the first months of the epidemic when the reproductive number was greater than one [5,18] and the epidemic was spreading quickly in communities. Furthermore, the level of knowledge about EVD varied over the course of the epidemic, as did communities’ sense of fear and/or distrust of EVD responders [9,10,20,21,23–27]. In such situations, each secondary case averted could be the first of many cases in an averted chain of transmission likely composed of multiple transmission generations and secondary cases. In Liberia, for example, one to seven transmission generations were documented in a single transmission chain with 4–35 secondary cases produced [14]. The estimates presented here correspond to the first transmission generation after the primary case and do not consider the impact that SDB had on chains of transmission averted by preventing cases beyond the first transmission generation. Consequently, the number of cases averted by SDB programs may be considerably higher than that which was directly estimated.

None of the investigations of unsafe burials of suspect EVD infected individuals reported here document a superspreading event, an event such as an unsafe burial that results in a disproportionately high number of secondary transmission (infection) to contacts. Nevertheless, SDB may have had an even greater impact on the epidemic by preventing superspreading events. Indeed, modeling suggests that such events may have sustained EVD transmission in Sierra Leone [28]. These events have been infrequently documented but have been known to occur after the death of an influential person in the community (e.g. elder or traditional healer), such as in Kissidougou (Guinea) where an unsafe burial of a male midwife assistant responsible for circumcisions in the community, was reported to have caused upwards of 85 secondary cases [29,30]. While superspreading events are relatively rare, if SDB prevented even one superspreading event, the impact on averted chains of transmission would be significant. Although such events were not identified during this research, both anecdotal reports and reports in peer reviewed literature suggest that superspreading events associated with unsafe burials did occur during the 2013–2016 outbreak [29,31].

The data presented here do have a number of limitations, including recall bias. As unsafe burials were investigated, the primary case was deceased and all information was collected from key informants. Age of the contact was frequently unknown and unable to be estimated with any certainty, particularly by key informants external to the family unit. Data were collected retrospectively and occasionally the unsafe burial investigated had occurred more than 12 months prior to the date of the interview, as was the case in Guéckédou (Guinea) and Kailahun (Sierra Leone). Conversely, as in Kambia and Western Area Rural (Sierra Leone), some unsafe burials had recently occurred and interviews with key informants were conducted while the individuals were still in quarantine. Frequently, key informants had themselves been infected with EVD and hospitalized due to the unsafe burial that was being investigated. In these cases it was difficult to collect data on the health outcomes of individuals who had participated in the unsafe burials if the outcome occurred during the hospitalization of the key informant. In some instances information could not be collected from familial key informants as they had died prior to the visit of the study teams. In such cases, additional key informants were identified. Furthermore, the risk factor analysis is limited by the amount of data that we were able to collect from key informants. Recall of caregiving and funeral practices was sometimes limited or not available. In addition, the status of some contacts, sick or EVD confirmed, was unknown if they did not return to their communities or share the information. In the absence of laboratory confirmation of EVD infection for all contacts reported to have become cases (fallen sick or had an EVD infection), we only documented the first transmission generation from the identified primary case. To the furthest extent possible, however, we attempted to triangulate the information collected during interviews in order to ensure the quality of the data and ensure it was exhaustive as possible. Finally, not all laboratory results were available or could be matched to the burials carried out, and the period for which results were available was limited.

Here, post-mortem transmission of EVD is investigated exclusively and the implications evaluated systematically for the first time. These findings underscore the substantial impact that SDB had on the 2013–2016 EVD epidemic with regards to the number of cases averted and the importance of early initiation of SDB activities during an EVD response. Previously, the importance of SDB as an integral part of reducing EVD transmission and stopping epidemics was acknowledged as a fundamental EVD control measure in terms of its impact on ending transmission, however its impact was not well understood.

The timing of SDB implementation and scale-up is also important and will affect the magnitude of the outbreak. The earlier in an EVD outbreak that a SDB program can be implemented at scale, the greater the number of cases averted and consequently the larger the program’s impact on the outbreak will be. Additional research is needed in order to better understand the variation of risk for EVD transmission related to distinct care practices both before and after death. The SDB program is a cornerstone of comprehensive EVD prevention and response programs and must be implemented as a holistic intervention incorporating both public health and socio-cultural perspectives in order to enable quick scale up and achieve maximum impact.

## Supporting information

S1 Checklist This file includes a checklist of information to include when reporting data from observational studies, indicating where specific items are reported in the paper. (DOC)
